# Molecular modeling analyses for graphene functionalized with Fe_3_O_4_ and NiO

**DOI:** 10.1016/j.heliyon.2020.e04456

**Published:** 2020-07-17

**Authors:** Amal H. Al-Bagawi, Ahmed M. Bayoumy, Medhat A. Ibrahim

**Affiliations:** a Chemistry Department, Faculty of Science, University of Ha’il, 1560, Hail, KSA; b Physics Department, Biophysics Branch, Faculty of Science, Ain Shams University, 11566, Cairo, Egypt; c Molecular Spectroscopy and Modeling Unit, Spectroscopy Department, National Research Centre, 33 El-Bohouth St., 12622 Dokki, Giza, Egypt

**Keywords:** Materials chemistry, Graphene, Molecular modeling, DFT, PM6, QSAR and MESP

## Abstract

Graphene has attracted great concern in recent years as one of the potential 2D materials in various applications. This work is devoted for assessing the feasibility of functionalizing 2D graphene sheets with ferromagnetic and antiferromagnetic metal oxides namely magnetite (Fe_3_O_4_) and nickel oxide (NiO). Molecular models of the proposed candidates are exposed to energy calculations at DFT level, in addition to geometry optimization processes at PM6 method. HOMO/LUMO orbitals, MESP maps and QSAR descriptors are calculated. Results ensure that graphene doped with NiO has the highest reactivity since it possesses the largest TDM and the smallest HOMO/LUMO band gap. MESP maps illustrate that the benzene rings of graphene are most probable to undergo nucleophilic interactions. Addition of Fe_3_O_4_ creates new negatively charged active sites that are ready for nucleophilic interactions. The calculated QSAR parameters demonstrate a hydrophobic nature for pure and modified graphene suggesting that they need further modification with further groups for usage in biological applications.

## 1 Introduction

Graphene, material composed of two dimensional one-atom-size plane of sp^2^-hybridized C-atoms in condensed packing honeycomb crystal structure, had witnessed significant attention representing the next-generation as electronic material due to its optimum properties especially its excellent current density, high thermal conductivity, ballistic transport, inert nature, optical transmittance and superhydrophobicity at nanoscale [1, 2]. It was firstly synthesized using graphite via micro-mechanical cleavage technique [3]. This approach allowed easy production of high-quality graphene crystallites and further led to enormous experimental activities [4] with the opposite magnetic properties. It is stated that, graphene was studied to be doped with various heteroatoms to obtain proper work functions for the field-emission [5]. While others reported its applications as high-performance catalytic activity [6] and dreaming electrode materials [7]. It was also functionalized with metal oxides to change the surface properties of graphene for wide applications such as nano-devices [8] and efficient catalysts for reactions [9]. Generally, nanotechnology always proves that it can control the assembling processes of different chemical structures at nanoscale materials [10]. Therefore, nanomaterials are often observed to be emerged among the focus of advanced research [11, 12]. Enhancements in various nanomaterials are not restricted to preparation techniques and characterizations, but also involve the theories concerned with interactions in such tiny scale [13, 14]. Recently, Fe_3_O_4_ magnetic nanoparticles (MNPs) have been intensively investigated due to their superparamagnetic characteristics, high coercivity and low Curie temperature [15, 16]. Beside these features, MNPs are also non-toxic and biocompatible. Therefore, Fe_3_O_4_ MNPs have been contributed into new trends for biomedical applications. Like dynamic sealing [17], biosensors [18], magnetic resonance (MR) imaging as a contrasting agent [19], therapeutic topical agent for hyperthermia [20] targeted-drug delivery system [21]. Nickel oxide (NiO) have attracted much attention due to its size-dependent crystal structure, vibration modes and magnetic properties, and its applications in catalysis, energy conversion, storage devices, battery electrodes, antiferromagnetic (AFM) layer, multidisciplinary sensors, electrochromic films, and transparent conducting films [22, 23]. Extensive studies have been reported in the beginning of this century on particle size-magnetic properties relations [24, 25, 26] and finite-size versus surface effects on magnetism [27], the fabulous effect of substitution [28] magnetism-matter crossover at ambient temperature [29, 30, 31, 32]. Molecular modelling methods is frequently utilized in order to investigate various molecular features such as structural, dynamical, geometrical, and thermodynamical properties of large number of structures [33, 34, 35]. These computational methods are currently used in order to model the molecular behavior to study physical, chemical as well as biological characteristics in several science branches and applications [36, 37, 38]. Molecular electrostatic potential (MESP) maps are one of the amazing features that can be investigated via molecular modeling. They are important in describing the active portions in chemical structures [39]. Such concept is useful since it determines the chemical addition nature through which a structure is most probable to have; either electrophilic or nucleophilic addition. Quantitative Structure Activity Relationship (QSAR) approach is another computational way that describes the unique relationship between a physicochemical characteristic of certain structure and its biological activity [40]. Some molecular parameters in terms of mathematical equations that clarify directly and/or indirectly its biological activity are obtained via QSAR calculations [41, 42]. Therefore, many researchers and several review articles continue to present QSAR descriptors [43, 44, 45].

Owing to the importance of functionalized grapheme, the present work is dedicate to model the graphene/Fe_3_O_4_ and graphene/NiO with Density Functional Theory (DFT) and PM6 semiemripcial method as well as QSAR descriptors.

## 2 Calculation details

All computations were carried out utilizing GAUSSIAN 09 software that is implemented at Spectroscopy Department, Physics Division, National Research Centre, NRC [46]. These calculations were performed via Density Functional Theory (DFT) level at Becke-Style 3-Parameter Density Functional Theory (using the Lee-Yang-Parr correlation functional) (B3LYP) [47, 48, 49] and 6-31G(d,p) basis set. After that, molecular electrostatic potential (MESP) maps are computed at the same method. Furthermore, physical parameters are extracted such as total energy (E), total dipole moment (TDM) and HOMO/LUMO band gap energy (ΔE).

Then, another optimization calculations were conducted using semiemprical quantum mechanical calculations at PM6 level [50] via SCIGRESS 3.0 software that is implemented at Spectroscopy Department, Physics Division, National Research Centre, NRC [51]. Finally, quantitative structure-activity relationship (QSAR) parameters such as Final heat of formation (FF), Ionization potential (IP), Log P, and Molar refractivity (MR) are also calculated at the same level. The long-range dispersion-correction was not used and the zero-point vibration energy was not also considered in the calculations.

## 3 Results and discussion

### 3.1 Building model molecules

**Figure 1:**
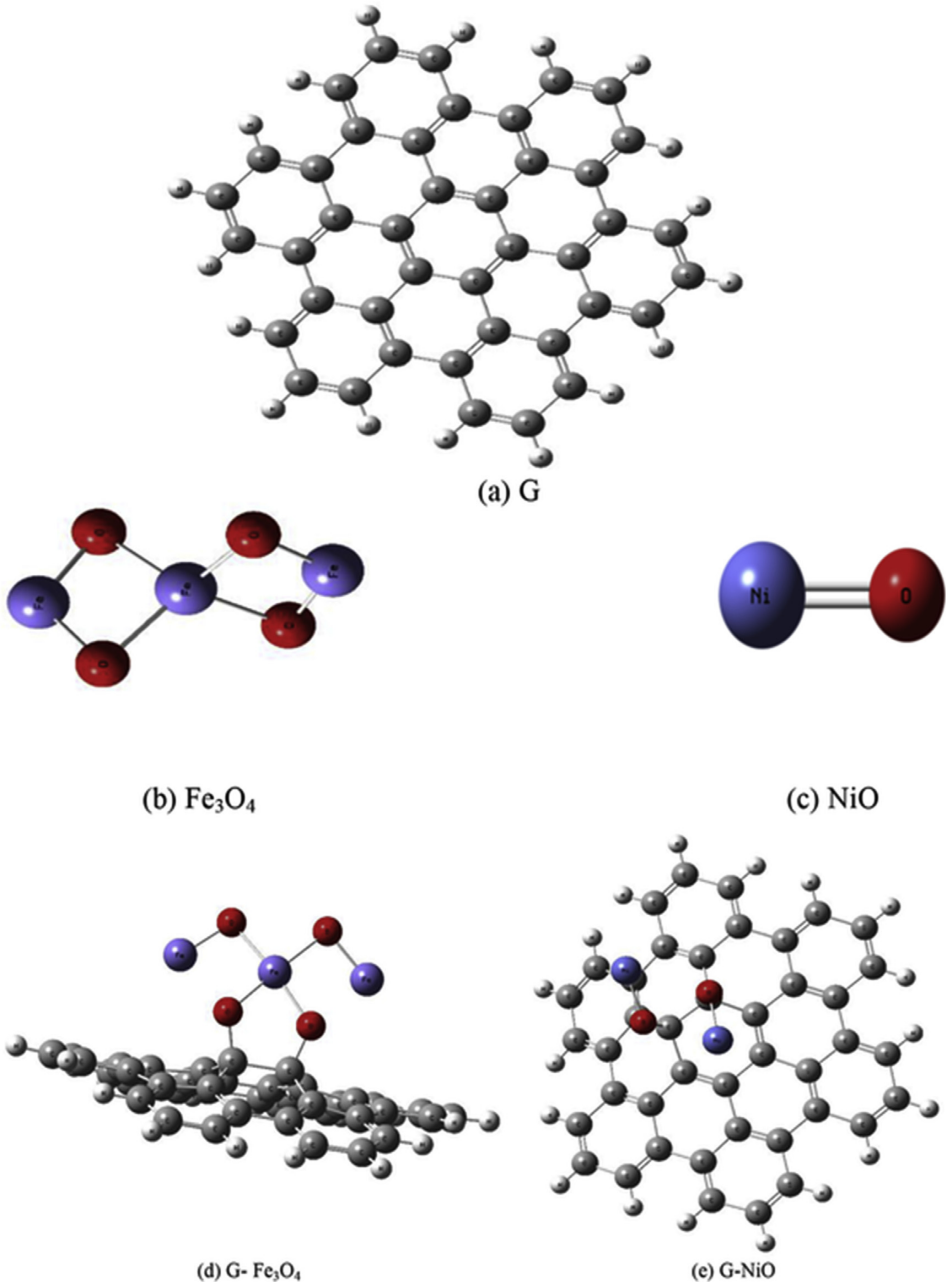
Model molecules of (a) graphene sheet (G), (b) magnetite (Fe_3_O_4_), (c) nickel oxide (NiO), (d) graphene functionalized with Fe_3_O_4_ (G- Fe_3_O_4_) and (e) graphene functionalized with NiO (G-NiO).

In order to describe the obtained results it is important to describe how the model molecules are built. Figure 1 demonstrates the constructed model molecules of graphene sheet (G), magnetite (Fe_3_O_4_), nickel oxide (NiO), graphene functionalized with Fe_3_O_4_ (G- Fe_3_O_4_), and graphene functionalized with NiO (G-NiO). Graphene is built as a 2D sheet composed of 42C atoms linked together with single and double bonds alternatively. Then, Fe_3_O_4_ and NiO are added to graphene sheet at nearly its central benzene ring; exactly at C16 and C17 atoms.

### 3.2 Energy calculations

As stated earlier in the calculation details section, the zero-point vibration energy is not considered in the calculations, and also the basis set superposition error (BSSE) has not been included in the energy calculations. Calculations were conducted to investigate the effect of functionalizing graphene with ferromagnetic substance (Fe_3_O_4_) and antiferromagnetic one (NiO) on both its electronic and physical features. These calculations are carried out using DFT theoretical level at B3LYP method and 6-31G(d,p) basis set. Table 1 presents the calculated physical parameters for the proposed structures such as total energy (E), total dipole moment (TDM) and HOMO/LUMO band gap energy (ΔE).

**Table 1:** Calculated physical parameters such as energy (E) as eV, total dipole moment (TDM) as Debye and band gap energy as eV for graphene sheet (G), magnetite (Fe_3_O_4_), nickel oxide (NiO), graphene functionalized with Fe_3_O_4_ (G- Fe_3_O_4_), and graphene functionalized with NiO (G-NiO) using B3LYP/6-31G(d,p) method.

Structure	E (keV)	TDM (Debye)	ΔE (eV)
G	-43.8459	0.0082	0.0928
NiO	-43.0809	3.9303	0.0799
Fe_3_O_4_	-111.3344	0.2309	0.0769
G-Fe_3_O_4_	-155.1801	5.5189	0.0711
G-NiO	-130.0085	8.9544	0.0040

Stability is one of the most important physical parameters that should be concerned when dealing with structures theoretically on the molecular scale. Total energy is the physical quantity which refers to stability level where the lower the structure's energy, the higher is its stability. The calculated energy of graphene sheet is about -43.8459 keV. NiO has a similar stability level to graphene while energy of Fe_3_O_4_ is much lower that of both graphene and NiO equaling to -111.3344 keV. Graphene functionalization with Fe_3_O_4_ and NiO results in structures of higher stability with energies -155.1801 and -130.0085 keV, respectively, indicating that addition of Fe_3_O_4_ makes graphene more stable than NiO. In addition, total dipole moment (TDM) is significant in determining structures' reactivity where those of large TDM values are considered more reactive relative to others. The resultant TDM of NiO is much larger than that of Fe_3_O_4_ with magnitudes 3.9303 and 0.2309 Debye, respectively reflecting higher reactivity for NiO with respect to Fe_3_O_4._ This can be attributed to that NiO has terminal reactive O atom while those of Fe_3_O_4_ are surrounded by Fe atoms. Therefore, TDM of graphene doped with NiO equals nearly one and half of that functionalized with Fe_3_O_4_. The calculated HOMO/LUMO band gap energies follow the same behavior of TDM ones ensuring the importance of band gap values in referring to the reactivity of chemical structures. The result of NiO is smaller than that of Fe_3_O_4_ and graphene functionalized with NiO possesses the smallest HOMO/LUMO band value of 0.0040 eV confirming its reactivity in comparison with the other proposed interaction with Fe_3_O_4_.

### 3.3 HOMO/LUMO calculations

**Figure 2:**
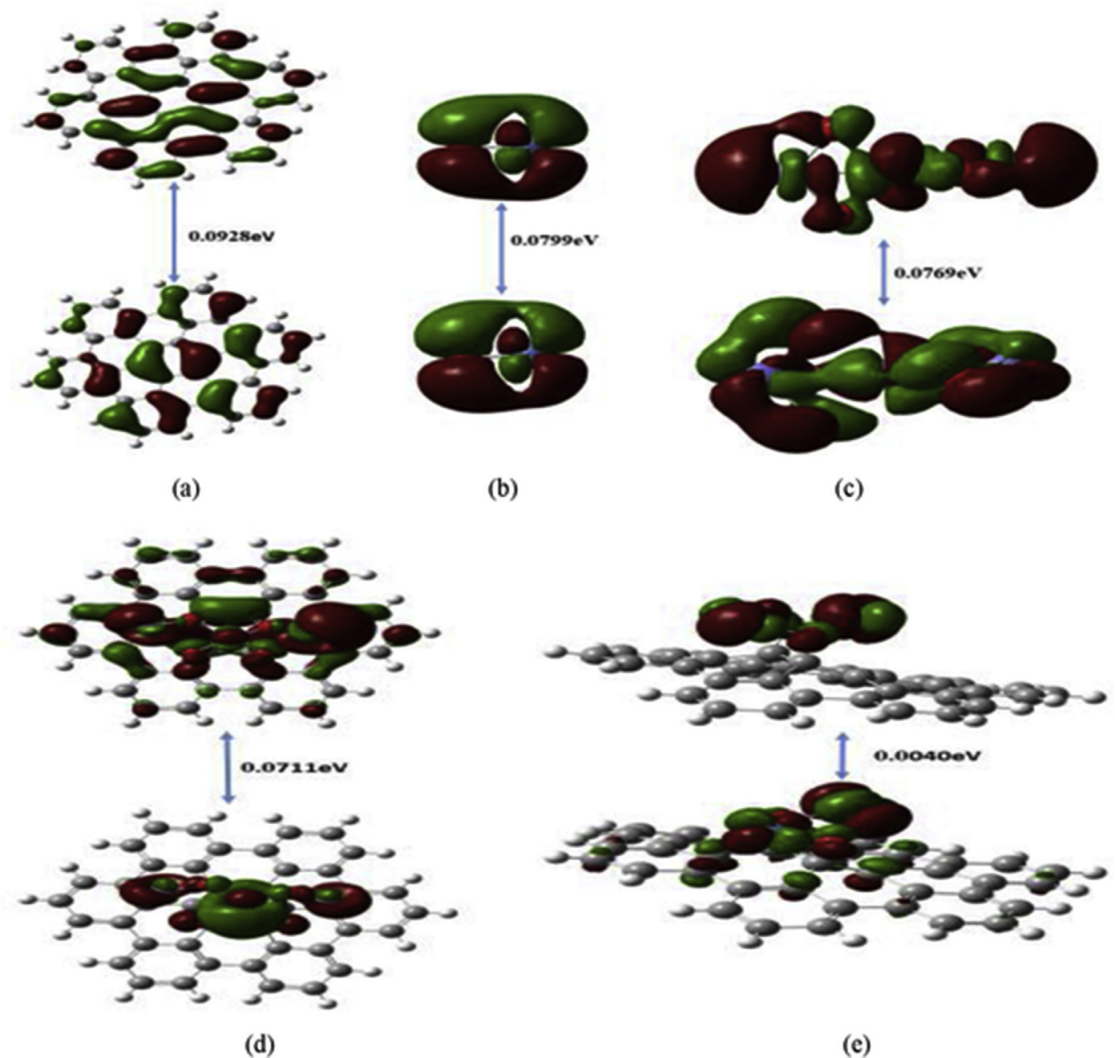
DFT Calculated HOMO/LUMO molecular orbitals of (a) graphene sheet (G), (b) magnetite (Fe_3_O_4_), (c) nickel oxide (NiO), (d) graphene functionalized with Fe_3_O_4_ (G- Fe_3_O_4_) and (e) graphene functionalized with NiO (G-NiO) at B3LYP/6-31G(d,p).

Figure 2 illustrates HOMO/LUMO orbitals of graphene sheet (G), magnetite (Fe_3_O_4_), nickel oxide (NiO), graphene functionalized with Fe_3_O_4_ (G-Fe_3_O_4_), and NiO (G-NiO) which computed at B3LYP/6-31G(d,p) theoretical level. Figures 2(a-e) map the HOMO and LUMO orbitals as separate figures showing the band gap energy required for an electron to transfer between HOMO and LUMO orbitals. HOMO/LUMO orbitals of graphene sheet, NiO and Fe_3_O_4_ show highly symmetric configurations indicating the homogeneity of electronic distribution through these orbitals either in the ground or excited states. However, they present asymmetric distribution for HOMO and LUMO of graphene functionalized with both Fe_3_O_4_ and NiO. For Fe_3_O_4__,_ the HOMO orbitals which are nearly concentrated around the added Fe_3_O_4_ group while the LUMO ones are distributed between both graphene sheet and Fe_3_O_4._ While the situation is reversed for graphene doped with NiO where the HOMO orbitals found in both entities and LUMO ones focused on NiO.

### 3.4 Molecular electrostatic potential (MESP) maps

**Figure 3:**
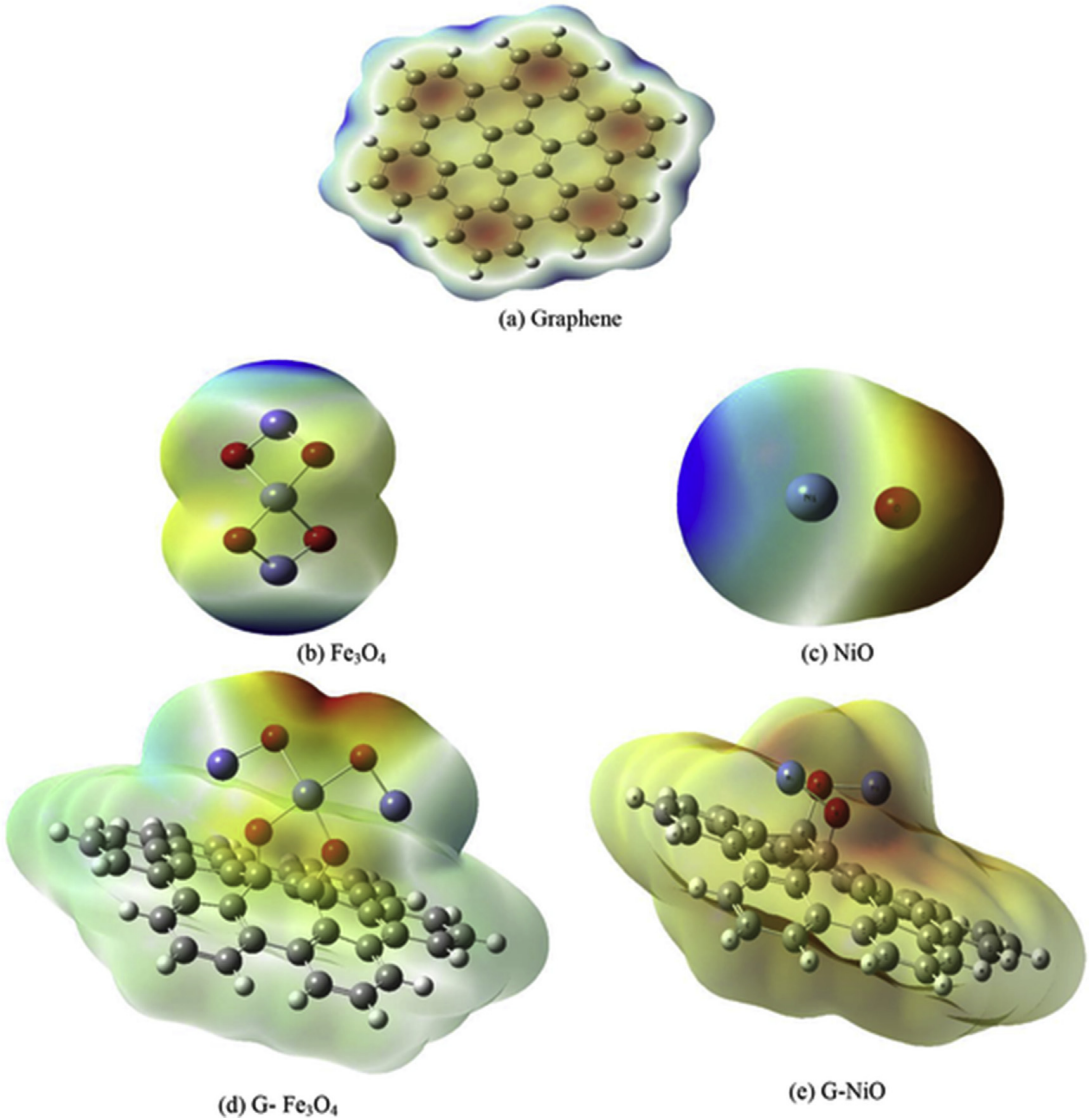
Calculated molecular electrostatic potential (MESP) maps of (a) graphene sheet (G), (b) magnetite (Fe_3_O_4_), (c) nickel oxide (NiO), (d) graphene functionalized with Fe_3_O_4_ (G- Fe_3_O_4_) and (e) graphene functionalized with NiO (G-NiO) on DFT using B3LYP/6-31G(d,p) method.

Molecular electrostatic potential (MESP) maps are created for the studied chemical structures at DFT level of theory using B3LYP/6-31G(d,p). They are usually provide simple and quite positive way for clarifying the charge distribution over a substance and so the most probable active sites in it. Figure 3 lists the constructed molecular electrostatic potential (MESP) maps for graphene (G), magnetite (Fe_3_O_4_), nickel oxide (NiO), graphene functionalized with Fe_3_O_4_ (G-Fe_3_O_4_) and NiO (G-NiO). The constructed maps composed of several colors extending from red color to dark blue one representing the extreme negative for red and positive sites for blue. These colors appear in the order of red, orange, yellow, green, light blue and dark blue from the most negative to most positive regions where red is for the extreme negative potentials and dark blue for positive ones. Furthermore, yellow regions have less negative potentials with respect to the red one and green reflects regions of neutral potentials. The potential distributions and colors as well can be correlated in a certain way by the electronegativity of the attached atoms. Atoms of high electronegativity seem to be red when linked to another less electronegative ones. Presence of atoms of nearly the same electronegativity makes colors distribution much narrower. Hence, we can depend on the MESP maps as a physical feature in deciding whether the interested active sites can undergo either nucleophilic or electrophilic chemical interactions.

Regarding MESP map of pure graphene sheet, there are three main colors composing the constructed map; red, blue and dark blue. It is noticeable that red is concentrated in the centers of benzene rings forming the sheet ensuring the effect of electron delocalization phenomenon within the rings suggesting that graphene core is most probable to undergo nucleophilic interactions. While the terminals are characterized by light and dark blue colors which may be attributed to presence of less electronegative H atoms. Hence, electrophilic reactions may be most likely to occur there. While magnetite structure is characterized by two main positive terminals due to the presence of Fe atoms colored by dark blue and nearly neutral central region colored by both yellow and light green. This may be due to the linkage of four O atoms to the central Fe atom canceling the effect of their high electronegativity. Such configuration propose that electrophilic chemical interaction is proper for magnetite ensuring the possibility of decorating graphene surface with it. NiO map contains two clear colors; red around O atom and blue surrounding Ni, reflecting the large difference in electronegativity between them. Thus, NiO may be involved in both electrophilic interactions utilizing Ni side and nucleophilic one from O terminal. Hence, NiO addition would be more convenient to interact with graphene sheet through Ni atom not oxygen. Turning to graphene functionalized with Fe_3_O_4_, the graphene appears with light blue color indicating positive region while the added magnetite has a range of colors; yellow around the O atoms linked to the sheet, light blue in the middle surrounding the central Fe nuclei and red in the vicinity of the upper O atoms indicating highly electronegative region. This reflects the success of oxygen electronegativity on withdrawing the electrons from graphene sheet. However, graphene linked to NiO structure is characterized by general yellow color except for certain light red regions on the upper surface where O atoms are present. This suggest that NiO addition turn the graphene to less negative structure.

### 3.5 QSAR descriptors

Quantitative structure-activity relationship (QSAR) descriptors provide a quite accessible and simple theoretical method for determining biological reactivity of chemical structures. QSAR always prove to be a point of research utilized when someone would like to assess biological activity [52, 53]. Its descriptors are calculated for the interested compounds at PM6 theoretical level. Table 2 presents the computed descriptors such as final heat of formation (FF), ionization potential (IP), Log P and molar refractivity (MR).

**Table 2:** PM6 computed QSAR parameters including final heat of formation (FF) as kcal/mol, ionization potential (IP) as eV, Log P and molar refractivity (MR) for graphene sheet (G), magnetite (Fe_3_O_4_), nickel oxide (NiO), graphene functionalized with Fe_3_O_4_ (G- Fe_3_O_4_) and NiO (G-NiO).

Structure	FF (kcal/mol)	IP (eV)	log P	MR
Graphene	146.972	-8.110	9.980	171.344
Fe_3_O_4_	-281.949	-10.687	1.085	5.401
NiO	154.042	-6.006	-0.351	1.443
G-Fe_3_O_4_	-84.614	-7.278	9.665	173.587
G-NiO	119.664	-7.782	9.122	170.887

For final heat of formation (FF) which is commonly known as the amount of heat change after the formation of a chemical substance of its individuals [54]. FF values are positive for graphene, NiO and graphene functionalized with NiO indicating that their formation processes followed by energy absorption [5]. While they are negative for Fe_3_O_4_ and G-Fe_3_O_4_ equaling -281.949 and -84.614 kcal/mol, respectively. Ionization potential or IP is the quantity of energy required to ionize a certain structure. It reflects structures' reactivity as well. Magnetite has the lowest IP value of -10.687 eV while NiO owns the largest one of -6.006 eV. Addition of either Fe_3_O_4_ or NiO to graphene increases its IP values. Partition coefficient or Log P is one of the QSAR biological descriptors that reflects the solubility of a substance in either organic or aqueous solvents. It is the ratio between the dissolved amounts in organic solvent to that does in aqueous one. Log P results indicate that all the proposed structures are hydrophobic except for NiO suggesting that they need further modification with further groups for usage in biological applications. Graphene is the most hydrophobic while Fe_3_O_4_ is the least one with values 9.98 and 1.085, respectively. Graphene functionalization slightly lowers its hydrophobicity. The computed molar refractivity (MR) of graphene, Fe_3_O_4_ and NiO equal 171.344, 5.401 and 1.443, respectively. Their interactions does not have significant impact on the MR of the resultant structures whose values are 173.587 for G-Fe_3_O_4_ and 170.887 for G-NiO.

## 4 Conclusion

Adding new functional groups for graphene is an important step towared directing its applications based on the functionalization process. Accordingly, it is aimed to assess the feasibility of functionalizing 2D graphene sheets with ferromagnetic (Fe_3_O_4_) and antiferromagnetic (NiO) materials in nanoscale. Based on DFT, PM6 and QSAR descriptors, one can drive some concluding remarks. Graphene doped with NiO has the highest reactivity since it possesses the largest TDM and the smallest HOMO/LUMO band gap. MESP maps illustrates that the benzene rings of graphene are most probable to undergo nucleophilic interactions. Addition of Fe_3_O_4_ to graphene create new negatively charged active sites that are ready for nucleophilic interactions as well. The calculated QSAR parameters demonstrate a hydrophobic nature for pure and modified graphene suggesting that they need further modification with further groups for usage in biological applications. Such results indicate that the reactivity of the given functionalized graphene can dedicate it for applications in the field of environmental pollution to control heavy metals for example from wastewater. This findings need some kind of experimental verifications which will be carried out in future based on the present computational study.

## Declarations

### Author contribution statement

Medhat Ibrahim, Amal H. Al-Bagawi, Ahmed M. Bayoumy: Conceived and designed the experiments; Performed the experiments; Analyzed and interpreted the data; Contributed reagents, materials, analysis tools or data; Wrote the paper.

### Funding statement

This research did not receive any specific grant from funding agencies in the public, commercial, or not-for-profit sectors.

### Competing interest statement

The authors declare no conflict of interest.

### Additional information

No additional information is available for this paper.
